# Prediction of Hematoma Expansion in Patients With Intracerebral Hemorrhage Using Thromboelastography With Platelet Mapping: A Prospective Observational Study

**DOI:** 10.3389/fneur.2021.746024

**Published:** 2021-10-15

**Authors:** Qiuguang He, You Zhou, Chang Liu, Zhongqiu Chen, Rong Wen, Yue Wu, Zongyi Xie, Yuan Cheng, Si Cheng

**Affiliations:** ^1^Department of Neurosurgery, The Second Affiliated Hospital of Chongqing Medical University, Chongqing, China; ^2^Department of Critical Care Medicine, The Second Affiliated Hospital of Chongqing Medical University, Chongqing, China; ^3^Department of Neurology, The Second Affiliated Hospital of Chongqing Medical University, Chongqing, China; ^4^Department of Information Center, The Second Affiliated Hospital of Chongqing Medical University, Chongqing, China; ^5^Department of Neurosurgery, The First Affiliated Hospital of Chongqing Medical University, Chongqing, China; ^6^Department of Orthopedics, The Second Affiliated Hospital of Chongqing Medical University, Chongqing, China

**Keywords:** intracerebral hemorrhage, hematoma expansion, thromboelastography, platelet function, stroke

## Abstract

**Background and Purpose:** The purpose of the study was to evaluate the usefulness of thromboelastography with platelet mapping (TEG-PM) for predicting hematoma expansion (HE) and poor functional outcome in patients with intracerebral hemorrhage (ICH).

**Methods:** Patients with primary ICH who underwent baseline computed tomography (CT) and TEG-PM within 6 h after symptom onset were enrolled in the observational cohort study. We performed univariate and multivariate logistic regression models to assess the association of admission platelet function with HE and functional outcome. In addition, a receiver operating characteristic (ROC) curve analysis investigated the accuracy of platelet function in predicting HE. A mediation analysis was undertaken to determine causal associations among platelet function, HE, and outcome.

**Results:** Of 142 patients, 37 (26.1%) suffered HE. Multivariate logistic regression identified arachidonic acid (AA) and adenosine diphosphate (ADP) inhibition as significant independent predictors of HE. The area under the ROC curves was 0.727 for AA inhibition and 0.721 for ADP inhibition. Optimal threshold for AA inhibition was 41.75% (75.7% sensitivity; 67.6% specificity) and ADP inhibition was 65.8% (73.0% sensitivity; 66.7% specificity). AA and ADP inhibition were also associated with worse 3-month outcomes after adjusting for age, admission Glasgow Coma Scale score, intraventricular hemorrhage, baseline hematoma volume, and hemoglobin. The mediation analysis showed that the effect of higher platelet inhibition with poor outcomes was mediated through HE.

**Conclusions:** These findings suggest that the reduced platelet response to ADP and AA independently predict HE and poor outcome in patients with ICH. Platelet function may represent a modifiable target of ICH treatment.

## Introduction

Spontaneous intracerebral hemorrhage (ICH) accounts for 20% of all strokes and carries the highest stroke-related morbidity and mortality (1, 2). Hematoma expansion (HE) usually occurs within the first few hours and is the main cause of worse functional outcome (3, 4). Therefore, therapeutic intervention aimed at reducing HE could represent a treatment paradigm in efforts to improve neurological outcome after ICH (2). However, recent ICH trials with hemostatic drugs, such as recombinant activated coagulation factor VII (rFVIIa) and tranexamic acid, did not reveal beneficial effects (5, 6). Previous studies focused on the changes of coagulation and fibrinolysis and theorized that these may contribute to HE. Actually, the role of platelet function on the occurrence of HE is insufficiently established, which may be due to the lack of effective detection methods (7).

Thromboelastography (TEG), a whole-blood viscoelastic test, offers a rapid bedside assessment of coagulability and fibrinolysis (8). Furthermore, according to the combined effect of platelet and plasma coagulation factors that contribute to hemostasis, platelet function activated by different pathways is available through TEG with platelet mapping (TEG-PM) (9). TEG-PM has previously been shown to be comparable with optical platelet aggregometry and superior to PFA-100 (10, 11). It is widely used in the guidance of personalized antiplatelet treatment and the assessment of perioperative period platelet function (11–13). However, there is no validated data about the level of platelet function detected by TEG-PM in patients with ICH.

The purpose of this prospective cohort study was to test the hypothesis that platelet dysfunction correlates with subsequent occurrence of HE and unfavorable outcome and then explore whether HE is the pathophysiological mechanism underlying this association. TEG-PM may be used as a clinically useful method to predict patients who will suffer HE and poor outcome and provide a possible direction concerning appropriate therapeutic interventions.

## Materials and Methods

### Patient Population

This prospective study included spontaneous ICH patients aged 18 years or older who were admitted to our center between November 2019 and February 2021. Inclusion criteria were as follows: (1) baseline computed tomography (CT) scan was obtained within 6 h of symptom onset and (2) follow-up CT scan was acquired within 24 h of the baseline CT scan.

Exclusion criteria were as follows: (1) the presence of vascular malformation, aneurysm, traumatic, brain tumor, ischemic stroke with hemorrhagic transformation, or any other cause of secondary ICH; (2) primary intraventricular hemorrhage; (3) preceding use of antiplatelet or anticoagulant drugs; (4) receiving any hemostatic agents before TEG-PM draw; (5) surgery or other neurosurgical intervention before follow-up CT scan; (6) evidence of coagulopathy on traditional laboratory testing (14), such as activated partial thromboplastin time (APTT) >50 s, international normalized ratio (INR) >1.5, or platelet (PLT) count <50 × 10^3^/μl; and (7) lost to follow-up. The Institutional Review Board of our hospital approved this study, and written informed consent was obtained from each patient or close relatives.

### Clinical Data and Outcome

We prospectively collected baseline demographic and clinical data including age, sex, cerebrovascular risk factors (smoking status, hypertension, and diabetes mellitus), onset to first CT scan time, admission systolic blood pressure (SBP), diastolic blood pressure (DBP), and baseline Glasgow Coma Scale (GCS) score at arrival. Laboratory information included hemoglobin (Hb), PLT, APTT, and INR.

All participants received standard care according to the ICH treatment protocol of our hospital. Systolic blood pressure target was less than 140 mmHg after admission, according to the American Heart Association/American Stroke Association guidelines (15). Functional outcome scored with the modified Rankin Scale (mRS) at 3 months was obtained by a trained research staff. Poor functional outcome was defined as a dichotomized mRS score of 3–6 according to previous studies (16–18).

### TEG-PM

TEG-PM testing was performed with whole blood drawn from a single clean puncture of the median cubital vein after the diagnostic CT scan. These samples were collected in heparinized or citrated tubes and processed within 2 h at room temperature. The tests were done using a computerized TEG-PM analyzer (Haemoscope, Model 5000) by a trained clinical scientist. Daily quality assurance checks were carried out to ensure the validity of calibration.

**Figure 1:**
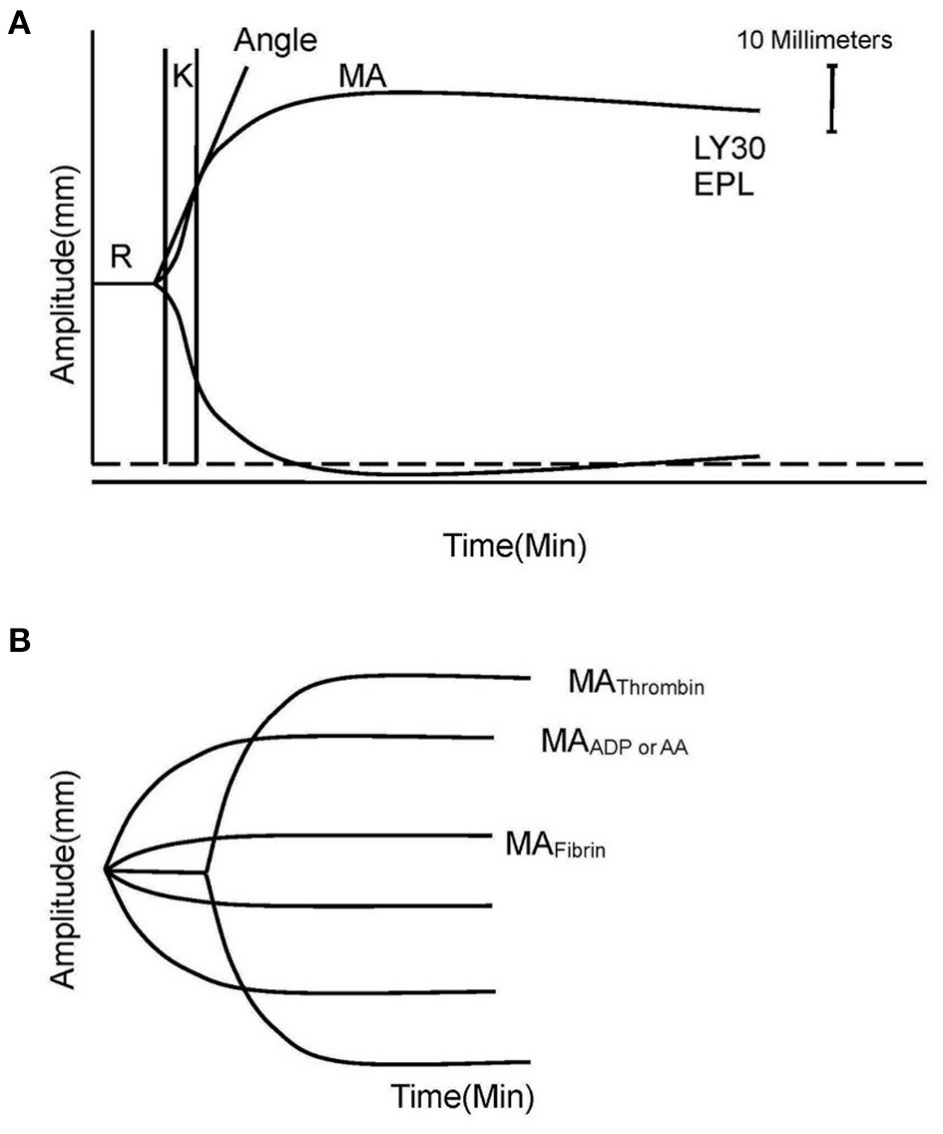
Explanation of TEG and TEG-PM. **(A)** TEG parameters: *K*, angle, MA, EPL, and LY30. **(B)** TEG-PM parameters: MA_Thrombin_, MA_ADP/AA_, and MA_Fibrin_. TEG, thromboelastography; TEG-PM, thromboelastography with platelet mapping; *R*, time from the start of the test to clot formation; *K*, kinetics of the rate of clot formation; angle, velocity of clot strength generation; MA, maximum amplitude; EPL, percent of clot dissolution 30 min after MA; LY30, percent of amplitude reduction at 30 min after MA; AA, arachidonic acid; ADP, adenosine diphosphate.

The standard TEG parameters were recorded as follows: time from the start of the test to clot formation (*R*, minutes), kinetics of the rate of clot formation (*K*, minutes), velocity of clot strength generation (angle, degrees), maximal clot strength contributed by fibrinogen activity and platelet function (MA, millimeters), percent of amplitude reduction at 30 min after MA (LY30), and percent of clot dissolution 30 min after MA (EPL) (Figure 1A) (8).

The platelet inhibition shows the degree of non-response of the platelet activated by exogenous arachidonic acid (AA) and adenosine diphosphate (ADP). MA_Thrombin_ reflects the maximal clot strength with contribution of platelet and fibrinogen together measured by a kaolin-activated sample. MA_AA_ and MA_ADP_ reflect clot strength induced by stimulation of AA or ADP, respectively. MA_Fibrin_ represents individual contribution of fibrin to the clot strength measured by a reptilase and activator F-activated sample. The platelet inhibitions in response to AA and ADP were calculated with computerized software according to the equation:


$$\begin{array}{l} 100-\{[(MA_{\text{AA}}orMA_{\text{ADP}})-MA_{\text{Fibrin}}]/\\ \;(MA_{\text{Thrombin}}-MA_{\text{Fibrin}})\}\times 100\; \end{array}$$

(Figure 1B) (19).

### Imaging Analysis

According to trial protocol, a follow-up CT scan was performed within 24 h after the admission CT. The interval from symptom onset to admission CT was recorded. All CT scans were analyzed to determine the presence of intraventricular hemorrhage, ICH location (infratentorial, lobar, or deep), and hematoma volume. Hematoma volume measurement was performed using the 3D Slicer. Regions of interest of hematoma were identified using a semiautomatic method in each slice with a threshold range from 44 to 100 Hounsfield units. Then, we calculated hematoma volume by the accumulating volume of each slice (20–22). HE was defined as relative growth of more than 33% or an absolute growth greater than 6 ml from initial CT according to previous studies (3, 8).

### Statistical Analysis

Quantitative variables were summarized as mean ± SD or as median with interquartile range (IQR) otherwise. Categorical variables were summarized as numbers with percentages. We compared baseline demographic, clinical, laboratory information, imaging characteristics, and TEG-PM parameters between patients with and without HE and then compared those between patients with good and poor outcome using *t* test (or Mann–Whitney *U* test for skewed distribution) for continuous variables and χ^2^ test (or Fisher's exact test) for categorical variables. To determine the independent predictors of HE and poor outcome, variables associated with a *P* < 0.10 in univariate analyses were entered into the multivariable logistic regression models. In addition, we performed a multivariable analysis in two models because GCS and baseline hematoma volume were collinear. A mediation analysis was performed to estimate whether HE (as the mediator) was the driving factor for any relationship between platelet inhibition (independent variable) and poor outcome (dependent variable) by regressing all three variables together (23).

To obtain diagnostic threshold values of inhibition, we used receiver operator characteristic (ROC) curves considering an area under the curve (AUC) of 0.70 or higher as indicating an acceptable prediction. From the ROC curve, we determined the optimal cut-off value (with sensitivity and specificity) for discriminating the risk of HE by maximizing the Youden index (24). Statistical significance was set at a *P* value < 0.05. Statistical analyses were performed using SPSS 19.0 software.

## Results

There were 142 ICH subjects meeting the inclusion criteria for analysis. The study population consists of 96 (67.6%) men and 46 (32.4%) women, with a mean age of 61.5 years. The median time from onset to first CT scan was 2.5 h (IQR, 2–4). The median baseline hematoma volume was 15 ml (IQR, 7–27 ml), and 48 patients (33.8%) had intraventricular hemorrhage on the first CT scan. HE occurred in 37 (26.1%) cases.

**Figure 2:**
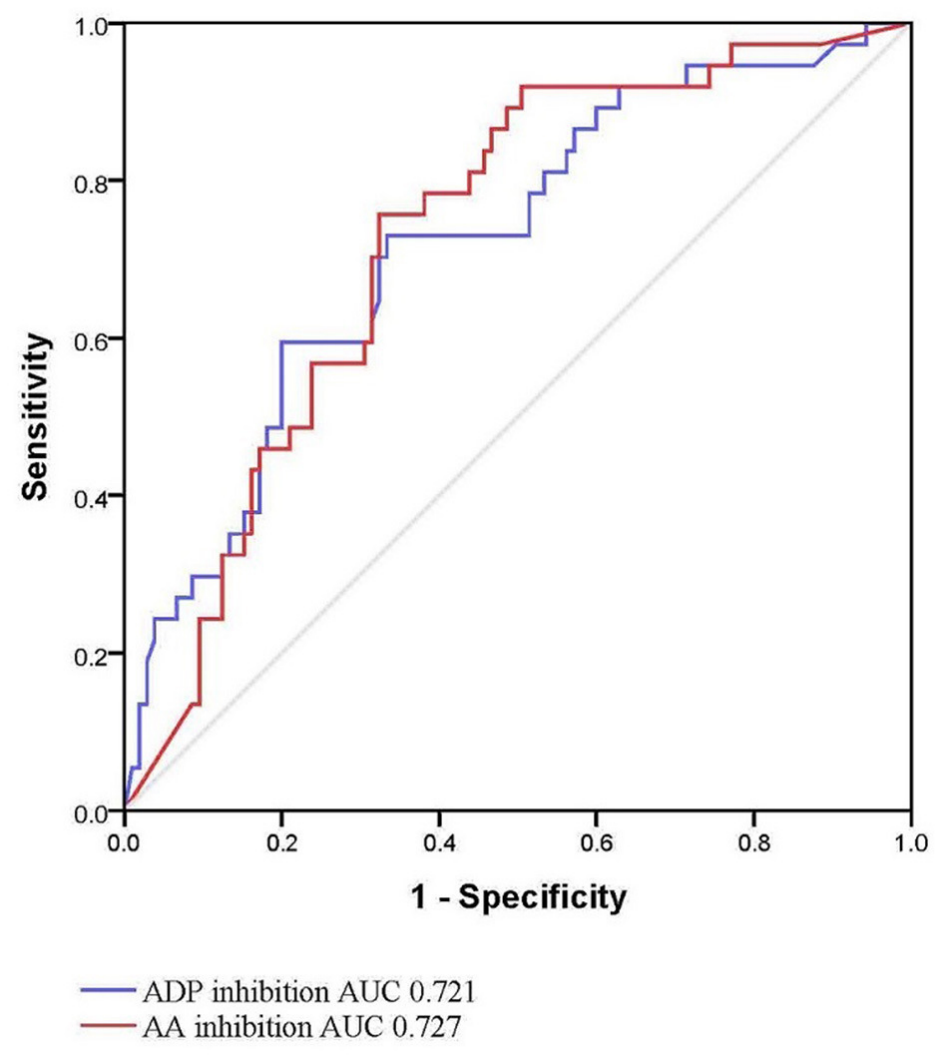
Receiver operating characteristic curve for hematoma expansion with AA and ADP inhibition. AA, arachidonic acid; ADP, adenosine diphosphate; AUC, area under the curve.

Intergroup differences between ICH patients with and without HE are shown in Table 1. Subjects with HE had higher systolic blood pressure, lower admission GCS score, shorter time to baseline CT scan, and larger baseline hematoma volume compared to subjects without HE (*P* < 0.05). There were no statistically significant differences in TEG values of *R, K*, angle, MA, EPL, and LY30 (*P* > 0.05). In multivariate logistic analyses adjusted for relevant confounders, independent predictors of HE were time to baseline CT scan, AA, and ADP inhibition in both models 1 and 2 (Table 2). The ability of AA and ADP inhibition to predict early HE is shown in Figure 2. The AUC were 0.727 (95% CI 0.638 to 0.816) for AA inhibition and 0.721 (95% CI 0.625 to 0.816) for ADP inhibition. We identified the optimal cut-off values of AA and ADP inhibition as 42.75% (sensitivity 75.7%; specificity 67.6%) and 65.8% (sensitivity 73.0%; specificity 66.7%) for predicting HE, respectively.

**Table 1:** Comparison of baseline demographic, clinical characteristics, and TEG-PM parameters between patients with and without hematoma expansion.

	**Hematoma expansion**		
	**Yes (*n* = 37)**	**No (*n* = 105)**	***P*** **value**
Male, *n* (%)	26 (70.3)	70 (66.7)	0.687
Age (years), mean ± SD	63.0 ± 14.5	61.0 ± 15.1	0.483
Smoking, *n* (%)	17 (45.9)	43 (41.0)	0.597
Hypertension, *n* (%)	31 (83.8)	86 (81.9)	0.796
Diabetes mellitus, *n* (%)	3 (8.1)	8 (7.6)	0.924
SBP on admission (mmHg), mean ± SD	184.5 ± 24.6	168.2 ± 31.2	0.005
DBP on admission (mmHg), mean ± SD	97.5 ± 17.1	97.8 ± 19.7	0.952
GCS score, median (IQR)	12 (8–14)	14 (12–15)	<0.001
Intraventricular hemorrhage, *n* (%)	15 (40.5)	33 (31.4)	0.314
Time to baseline CT scan (h), median (IQR)	2 (1–2.5)	3 (2–4.5)	<0.001
Baseline hematoma volume (ml), median (IQR)	22 (10–34.5)	14 (4.5–26)	0.007
**ICH locations**			0.846
Infratentorial, *n* (%)	3 (8.1)	8 (7.6)	
Lobar, *n* (%)	9 (24.3)	21 (20.0)	
Deep, *n* (%)	25 (67.6)	76 (72.4)	
Hb (g/l), mean ± SD	136.3 ± 17.0	139.1 ± 18.1	0.412
PLT count (1,000 cells/μl), mean ± SD	196.1 ± 52.7	192.5 ± 59.9	0.750
APTT (s), median (IQR)	34.9 (32.1–38.5)	34.8 (32.4–37.2)	0.672
INR, median (IQR)	1.02 (0.96–1.07)	1.03 (0.97–1.12)	0.262
*R* (min), mean ± SD	6.7 ± 2.6	6.3 ± 1.7	0.446
*K* (min), median (IQR)	1.9 (1.6–2.9)	1.8 (1.6–2.3)	0.153
Angle (°), median (IQR)	62.1 (55.3–67.5)	64.4 (58.2–68.3)	0.192
MA (mm), mean ± SD	59.0 ± 6.8	60.9 ± 5.9	0.111
EPL (%), median (IQR)	0.1 (0.1–1.2)	0.2 (0.1–2.1)	0.108
LY30 (%), median (IQR)	0.1 (0.1–0.5)	0.1 (0.1–0.9)	0.359
AA inhibition (%), median (IQR)	77.9 (38.9–98.8)	23.5 (8.9–72.0)	<0.001
ADP inhibition (%), median (IQR)	80.3 (47.5–95.8)	51.5 (21.6–72.2)	<0.001

*TEG-PM, thromboelastography with platelet mapping; SD, standard deviation; SBP, systolic blood pressure; DBP, diastolic blood pressure; GCS, Glasgow Coma Scale; CT, computed tomography; ICH, intracerebral hemorrhage; Hb, hemoglobin; PLT, platelet; APTT, activated partial thromboplastin time; IQR, interquartile range; INR, international normalized ratio; R, time from the start of the test to clot formation; K, kinetics of the rate of clot formation; Angle, velocity of clot strength generation; MA, maximum amplitude; EPL, percent of clot dissolution 30 min after MA; LY30, percent of amplitude reduction at 30 min after MA; AA, arachidonic acid; ADP, adenosine diphosphate*.

**Table 2:** Multivariate analysis of predictors for hematoma expansion.

	**OR**	**95% Wald CI**	***P*** **value**
**Model 1**			
SBP on admission, mmHg	1.016	0.999–1.032	0.061
Time to baseline CT scan, h	0.575	0.417–0.793	0.001
Baseline hematoma volume, ml	1.014	0.987–1.041	0.327
AA inhibition, %	1.020	1.006–1.033	0.004
ADP inhibition, %	1.026	1.008–1.045	0.004
**Model 2**			
SBP on admission, mmHg	1.012	0.995–1.029	0.167
Time to baseline CT scan, h	0.588	0.424–0.815	0.001
GCS score	0.861	0.732–1.013	0.072
AA inhibition, %	1.022	1.008–1.035	0.001
ADP inhibition, %	1.025	1.006–1.043	0.008

*OR, odds ratio; CI, confidence interval; SBP, systolic blood pressure; GCS, Glasgow Coma Scale; CT, computed tomography; AA, arachidonic acid; ADP, adenosine diphosphate*.

Eighty-eight patients (62.0%) with ICH had unfavorable outcome. The results of bivariate analysis concerning predictors of 3-month outcomes are detailed in Table 3. The age, admission GCS score, intraventricular hemorrhage, baseline hematoma volume, and AA and ADP inhibition were associated with unfavorable outcome. The multivariate logistic analysis showed that the AA and ADP inhibition remained independent predictors of unfavorable outcome in patients with ICH (Table 4).

**Table 3:** Univariate comparison between patients with good outcome (mRS 0–2) and poor outcome (mRS 3–6).

	**Good outcome (*n* = 54)**	**Poor outcome (*n* = 88)**	***P*** **value**
Male, *n* (%)	37 (68.5)	59 (67.0)	0.856
Age (years), mean ± SD	56.2 ± 12.0	64.8 ± 15.6	<0.001
Smoking, *n* (%)	24 (44.4)	36 (40.9)	0.679
Hypertension, *n* (%)	43 (79.6)	74 (84.1)	0.498
Diabetes mellitus, *n* (%)	4 (7.4)	7 (8.0)	0.906
SBP on admission (mmHg), mean ± SD	171.1 ± 32.0	173.3 ± 29.6	0.680
DBP on admission (mmHg), mean ± SD	101.1 ± 21.0	95.6 ± 17.4	0.110
GCS score, median (IQR)	14 (13–15)	13 (9–14)	<0.001
Intraventricular hemorrhage, *n* (%)	12 (22.2)	36 (40.9)	0.022
Time to baseline CT scan (h), median (IQR)	2 (2–4)	3 (1.25–4)	0.959
Baseline hematoma volume (ml), median (IQR)	7 (3–15)	22.5 (12–34.5)	<0.001
**ICH locations**			0.216
Infratentorial, *n* (%)	6 (11.1)	5 (5.7)	
Lobar, *n* (%)	8 (14.8)	22 (25.0)	
Deep, *n* (%)	40 (74.1)	61 (69.3)	
Hb (g/l), mean ± SD	141.5 ± 16.6	136.4 ± 18.3	0.095
PLT count (1,000 cells/μl), mean ± SD	196.7 ± 56.0	191.5 ± 59.3	0.606
APTT (s), median (IQR)	34.9 (32.6–37.1)	34.7 (32.0–37.5)	0.621
INR, median (IQR)	1.04 (0.97–1.11)	1.02 (0.97–1.10)	0.357
*R* (min), mean ± SD	6.3 ± 1.4	6.5 ± 2.2	0.456
*K* (min), median (IQR)	1.8 (1.6–2.3)	1.9 (1.6–2.8)	0.544
Angle (°), median (IQR)	65.6 (59.0–68.2)	63.5 (54.6–67.7)	0.242
MA (mm), mean ± SD	61.4 ± 6.6	59.8 ± 5.8	0.130
EPL (%), median (IQR)	0.1 (0.1–1.3)	0.2 (0.1–2.0)	0.329
LY30 (%), median (IQR)	0.1 (0.1–0.5)	0.1 (0.1–1.0)	0.162
AA inhibition (%), median (IQR)	18.5 (6.6–33.1)	56.3 (23.9–94.6)	<0.001
ADP inhibition (%), median (IQR)	38.6 (16.7–63.2)	69.3 (39.7–89.4)	<0.001

*mRS, modified Rankin Scale; SD, standard deviation; SBP, systolic blood pressure; DBP, diastolic blood pressure; GCS, Glasgow Coma Scale; CT, computed tomography; ICH, intracerebral hemorrhage; Hb, hemoglobin; PLT, platelet; APTT, activated partial thromboplastin time; IQR, interquartile range; INR, international normalized ratio; R, time from the start of the test to clot formation; K, kinetics of the rate of clot formation; Angle, velocity of clot strength generation; MA, maximum amplitude; EPL, percent of clot dissolution 30 min after MA; LY30, percent of amplitude reduction at 30 min after MA; AA, arachidonic acid; ADP, adenosine diphosphate*.

**Table 4:** Multivariate analysis of predictors for poor outcome.

	**OR**	**95% Wald CI**	***P*** **value**
**Model 1**			
Age, years	1.070	1.029–1.112	0.001
Intraventricular hemorrhage	1.638	0.540–4.968	0.383
Baseline hematoma volume, ml	1.144	1.078–1.214	<0.001
Hb, g/l	1.006	0.977–1.036	0.694
AA inhibition, %	1.019	1.005–1.033	0.007
ADP inhibition, %	1.027	1.010–1.045	0.002
**Model 2**			
Age, years	1.045	1.012–1.080	0.007
GCS score	0.679	0.538–0.858	0.001
Intraventricular hemorrhage	1.710	0.627–4.664	0.294
Hb, g/l	1.000	0.974–1.026	0.973
AA inhibition, %	1.021	1.008–1.034	0.002
ADP inhibition, %	1.021	1.005–1.036	0.009

*OR, odds ratio; CI, confidence interval; GCS, Glasgow Coma Scale; Hb, hemoglobin; AA, arachidonic acid; ADP, adenosine diphosphate*.

**Figure 3:**
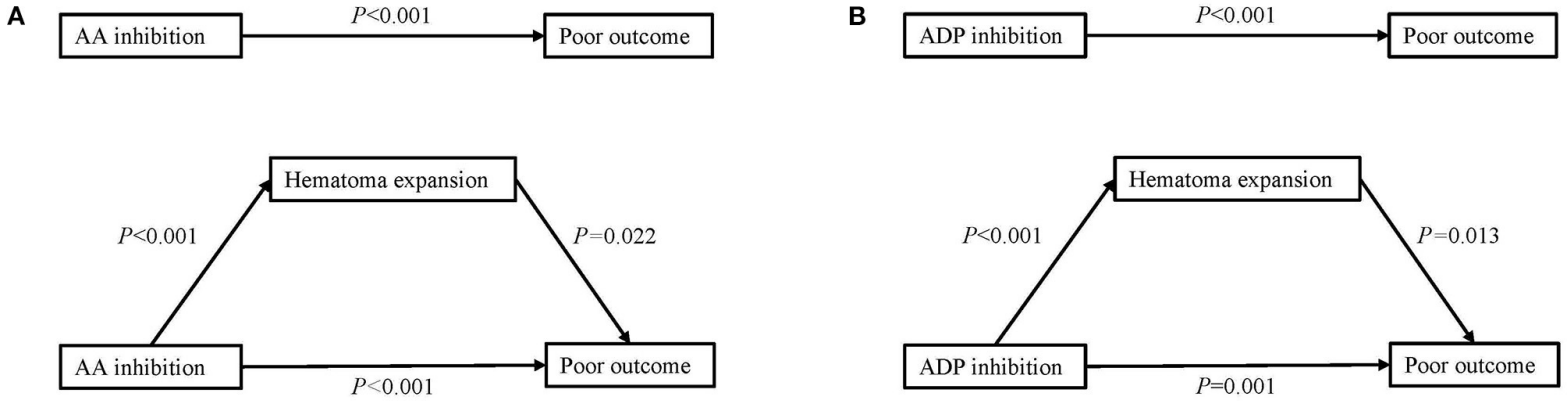
Mediation analysis of the association of platelet inhibition with poor outcome with hematoma expansion as a mediator. **(A)** Mediation analysis of AA inhibition in poor outcome by hematoma expansion. **(B)** Mediation analysis of ADP inhibition in poor outcome by hematoma expansion. AA, arachidonic acid; ADP, adenosine diphosphate.

When regressing platelet inhibition, HE, and outcome together, the mediation analysis revealed that HE partially mediated the relationship between a high degree of AA inhibition and poor outcome. Additionally, HE also played a partially mediating role between ADP inhibition and poor outcome (Figure 3).

## Discussion

Our data indicate that a higher degree of AA and ADP inhibition on admission was independently associated with increased odds of HE. Furthermore, AUC suggested the acceptable performance of platelet function to predict HE. In addition, our study demonstrated that admission platelet dysfunction was associated with poor outcome at 3 months, after adjustment for age and measures of disease severity. This relationship seemed to be mediated partly by HE.

A previous study revealed that patients with HE showed longer *K* and a trend toward longer *R* compared with patients without HE (8). However, we were unable to identify an association of coagulation status with HE detected by TEG-PM. These differences may have been explained by the inclusion of ICH patients receiving anticoagulant or antiplatelet therapy in that study, and there were obviously more patients receiving aspirin and clopidogrel in the HE group, thus causing a slower clot formation. Additionally, the prior study did not include information on times from symptom onset to admission CT, a well-known predictor of HE. What is more, GCS score and baseline hematoma volume were not associated with HE in that study, which is not consistent with previously reported ICH cohorts (3, 25).

Antiplatelet medication has been demonstrated to be related to increased risk of HE and poor prognosis, due to the platelet dysfunction (26). Actually, high variability of platelet reactivity to ADP and AA in healthy volunteers has previously been reported (11, 27), but few studies concerned about the relationship between platelet function and HE in ICH patients without antiplatelet therapy before. In our cohort, the degree of ADP and AA receptor inhibition was similar to the clinical scenario of patients taking aspirin combined with clopidogrel (11). It is unknown whether ICH itself changes platelet activity, because it is not feasible to obtain platelet activity data before and after ICH in humans. Furthermore, our multivariable analysis suggested that both AA and ADP inhibition are independent predictors of HE. However, no difference was demonstrated in platelet count, coagulation, or fibrinolysis status between the two groups.

Although our study was not designed to investigate potential pathophysiologic mechanisms of platelet function in HE, multiple prior studies support a definitely mechanistic role for platelet function in hemostasis. When bleeding occurs, the platelet and fibrin polymer aggregate in the damaged vessel to form an immobile blood clot. Secondary mechanical shearing of peripheral vessels caused by the initial bleeding is responsible for ongoing bleeding, called HE (25, 28). Otherwise, the accumulation of platelets activated through AA and ADP pathway subsequently enhance the hemostatic effect to prevent HE (29). This could be a possible explanation of the present study that HE is more likely to occur in patients with high ADP or AA inhibition.

According to the role of platelet in hemostasis, we speculate that the effect of higher platelet inhibition on outcomes in ICH patients is mediated through larger admission hematoma volumes and HE. This in turn results in worse functional outcomes at 3 months. In the mediation analysis, we were able to demonstrate a HE-mediated mechanism contributing to the association between platelet inhibition and functional outcome. Moreover, it is possible that mechanisms unrelated to HE may also contribute to the association.

HE is a clear independent predictor of increased mortality and poor functional outcomes (17). Therefore, several agents that affect the coagulation and fibrinolysis status have been investigated to prevent HE, restricting the mass effect and secondary brain injury (30). Unfortunately, none of these clinical trials was able to reject the null hypothesis (31). In previous studies, tranexamic acid and rFVIIa reduced HE compared with placebo, but did not improve survival or functional outcomes (6, 32). Our data may suggest that improvement of platelet function can be considered in preventing the early occurrence of HE to improve outcome. However, a previous study showed that platelet transfusion did not reduce HE for people taking antiplatelet therapy before ICH. On the contrary, it was associated with increased mortality and dependence in 3 months (18). One possible explanation is that HE is more closely associated with platelet function than platelet count itself, as demonstrated in our multivariable analysis and a previous cohort study (33). Another possible explanation is that the harmful effects were partly caused by transfusing ABO-incompatible platelets (34). Two prior studies have investigated the effect of desmopressin on improving platelet function in ICH patients and found that intravenous desmopressin was well tolerated and obviously improved platelet activity (35, 36). According to the results of our study, it is reasonable to believe that desmopressin may be a potential pharmacological treatment in preventing HE to improve outcome.

Thus far, clinical trials aimed at restricting expansion have not led to improved neurological outcome (6, 18), perhaps because such therapies need to be targeted to patients at highest risk for expansion to show any benefit. So, effective biomarkers are needed to select patients and guide interventions to restrict HE. The computed tomographic angiography (CTA) spot sign, a novel radiographic marker, has proven to be a promising predictor of HE with a sensitivity of 51% and a specificity of 85% (3). In our study, the sensitivity and specificity of platelet inhibition for predicting expansion were 73.0 and 66.7% for ADP inhibition and 75.7 and 67.6% for AA inhibition, respectively. Compared with the CTA spot sign, platelet inhibition has a lower specificity, but it is more sensitive for predicting HE. Moreover, CTA spot sign was just an imaging sign that cannot be altered, while platelet function can be directly improved. Additionally, recent clinical trials did not show reduced HE or improved clinical outcomes in spot sign-positive ICH patients through the use of rFVIIa or tranexamic acid (37, 38). Thus, it may be infeasible to use CTA spot sign as a predictor to target hemostatic therapy in acute ICH patients. Our data may suggest that TEG-PM is useful to identify individuals in high risk for HE, and they should receive aggressive treatments such as desmopressin and hemostatic drugs.

Inherent limitations of our analysis should be clarified. Our study was a single-center observational cohort with limited sample size and will need to be replicated. In addition, TEG-PM was tested only once on admission. It is unclear whether there are dynamical changes in platelet function, which might explain the reason why HE often occurs in early stage. Finally, we did not perform CTA spot sign testing and the correlation of platelet function with the CTA spot sign was not analyzed.

Further investigation is warranted to confirm our findings of more HE after ICH in patients with lower platelet function, resulting in worse clinical outcomes. If confirmed, these findings may suggest the importance of accounting for and correcting platelet dysfunction in future ICH treatment strategies for HE in efforts to improve outcome.

## Data Availability Statement

The raw data supporting the conclusions of this article will be made available by the authors, without undue reservation.

## Ethics Statement

The studies involving human participants were reviewed and approved by Medical Ethics Committee of the Second Affiliated Hospital of Chongqing Medical University. The patients/participants provided their written informed consent to participate in this study.

## Author Contributions

QH, YC, and SC conceived and designed the study. QH, YZ, CL, RW, and YW acquired the data, which ZC analyzed. QH, YZ, and ZX aided in data interpretation and wrote the manuscript. All authors contributed to the article and approved the submitted version.

## Funding

This work was supported by Chongqing Medical Scientific Research Project (No. 2022WSJK036).

## Conflict of Interest

The authors declare that the research was conducted in the absence of any commercial or financial relationships that could be construed as a potential conflict of interest.

## Publisher's Note

All claims expressed in this article are solely those of the authors and do not necessarily represent those of their affiliated organizations, or those of the publisher, the editors and the reviewers. Any product that may be evaluated in this article, or claim that may be made by its manufacturer, is not guaranteed or endorsed by the publisher.

## References

[1] Qureshi AI Mendelow AD Hanley DF. Intracerebral haemorrhage. Lancet. (2009) 373:1632–44. 10.1016/S0140-6736(09)60371-8 19427958 PMC3138486

[2] Keep RF Hua Y Xi G. Intracerebral haemorrhage: mechanisms of injury and therapeutic targets. Lancet Neurol. (2012) 11:720–31. 10.1016/S1474-4422(12)70104-7 22698888 PMC3884550

[3] Demchuk AM Dowlatshahi D Rodriguez-Luna D Molina CA Blas YS Dzialowski I. Prediction of haematoma growth and outcome in patients with intracerebral haemorrhage using the CT-angiography spot sign (PREDICT): a prospective observational study. Lancet Neurol. (2012) 11:307–14. 10.1016/S1474-4422(12)70038-8 22405630

[4] Davis SM Broderick J Hennerici M Brun NC Diringer MN Mayer SA. Hematoma growth is a determinant of mortality and poor outcome after intracerebral hemorrhage. Neurology. (2006) 66:1175–81. 10.1212/01.wnl.0000208408.98482.99 16636233

[5] Al-Shahi Salman R Law ZK Bath PM Steiner T Sprigg N. Haemostatic therapies for acute spontaneous intracerebral haemorrhage. Cochrane Datab Systemat Rev. (2018) 4:CD005951. 10.1002/14651858.CD005951.pub4 29664991 PMC6494564

[6] Sprigg N Flaherty K Appleton JP Al-Shahi Salman R Bereczki D Beridze M. Tranexamic acid for hyperacute primary IntraCerebral Haemorrhage (TICH-2): an international randomised, placebo-controlled, phase 3 superiority trial. Lancet. (2018) 391:2107–15. 10.1016/S0140-6736(18)31033-X 29778325 PMC5976950

[7] Burchell SR Tang J Zhang JH. Hematoma expansion following intracerebral hemorrhage: mechanisms targeting the coagulation cascade and platelet activation. Curr Drug Targets. (2017) 18:1329–44. 10.2174/1389450118666170329152305 28378693 PMC6894484

[8] Kawano-Castillo J Ward E Elliott A Wetzel J Hassler A McDonald M. Thrombelastography detects possible coagulation disturbance in patients with intracerebral hemorrhage with hematoma enlargement. Stroke. (2014) 45:683–8. 10.1161/STROKEAHA.113.003826 24425123 PMC4115455

[9] He Q Zhou Y Liu C Zhang X Huang N Wang F. Thromboelastography with platelet mapping detects platelet dysfunction in patients with aneurysmal subarachnoid hemorrhage with rebleeding. Neuropsychiatr Dis Treat. (2019) 15:3443–51. 10.2147/NDT.S229284 31908459 PMC6924584

[10] Agarwal S Coakley M Reddy K Riddell A Mallett S. Quantifying the effect of antiplatelet therapy: a comparison of the platelet function analyzer (PFA-100) and modified thromboelastography (mTEG) with light transmission platelet aggregometry. Anesthesiology. (2006) 105:676–83. 10.1097/00000542-200610000-00011 17006064

[11] Collyer TC Gray DJ Sandhu R Berridge J Lyons G. Assessment of platelet inhibition secondary to clopidogrel and aspirin therapy in preoperative acute surgical patients measured by thrombelastography platelet mapping. Br J Anaesth. (2009) 102:492–8. 10.1093/bja/aep039 19286767

[12] Kasivisvanathan R Abbassi-Ghadi N Kumar S Mackenzie H Thompson K James K. Risk of bleeding and adverse outcomes predicted by thromboelastography platelet mapping in patients taking clopidogrel within 7 days of non-cardiac surgery. Br J Surg. (2014) 101:1383–90. 10.1002/bjs.9592 25088505

[13] Zhao X Li Q Tu C Zeng Y Ye Y. High glycated albumin is an independent predictor of low response to clopidogrel in ACS patients: a cross-sectional study. Cardiovasc Diabetol. (2020) 19:171. 10.1186/s12933-020-01146-w 33036613 PMC7545941

[14] Roh D Torres GL Cai C Zammit C Reynolds AS Mitchell A. Coagulation differences detectable in deep and lobar primary intracerebral hemorrhage using thromboelastography. Neurosurgery. (2020) 87:918–24. 10.1093/neuros/nyaa056 32167143

[15] Hemphill JC 3rd Greenberg SM Anderson CS Becker K Bendok BR Cushman M. Guidelines for the management of spontaneous intracerebral hemorrhage: a guideline for healthcare professionals from the American Heart Association/American Stroke Association. Stroke. (2015) 46:2032–60. 10.1161/STR.0000000000000069 26022637

[16] Anderson CS Heeley E Huang Y Wang J Stapf C Delcourt C. Rapid blood-pressure lowering in patients with acute intracerebral hemorrhage. N Engl J Med. (2013) 368:2355–65. 10.1056/NEJMoa1214609 23713578

[17] Selim M Foster LD Moy CS Xi G Hill MD Morgenstern LB. Deferoxamine mesylate in patients with intracerebral haemorrhage (i-DEF): a multicentre, randomised, placebo-controlled, double-blind phase 2 trial. Lancet Neurol. (2019) 18:428–38. 10.1016/S1474-4422(19)30069-9 30898550 PMC6494117

[18] Baharoglu MI Cordonnier C Al-Shahi Salman R de Gans K Koopman MM Brand A. Platelet transfusion versus standard care after acute stroke due to spontaneous cerebral haemorrhage associated with antiplatelet therapy (PATCH): a randomised, open-label, phase 3 trial. Lancet. (2016) 387:2605–13. 10.1016/S0140-6736(16)30392-0 27178479

[19] Gurbel PA Bliden KP Tantry US Monroe AL Muresan AA Brunner NE. First report of the point-of-care TEG: a technical validation study of the TEG-6S system. Platelets. (2016) 27:642–9. 10.3109/09537104.2016.1153617 27809712

[20] Xu X Chen X Li F Zheng X Wang Q Sun G. Effectiveness of endoscopic surgery for supratentorial hypertensive intracerebral hemorrhage: a comparison with craniotomy. J Neurosurg. (2018) 128:553–9. 10.3171/2016.10.JNS161589 28387618

[21] Chen M Li Z Ding J Lu X Cheng Y Lin J. Comparison of common methods for precision volume measurement of hematoma. Comput Math Methods Med. (2020) 2020:6930836. 10.1155/2020/6930836 32724331 PMC7382736

[22] Xu X Chen X Zhang J Zheng Y Sun G Yu X. Comparison of the Tada formula with software slicer: precise and low-cost method for volume assessment of intracerebral hematoma. Stroke. (2014) 45:3433–5. 10.1161/STROKEAHA.114.007095 25316277

[23] Preacher KJ Hayes AF. SPSS and SAS procedures for estimating indirect effects in simple mediation models. Behav Res Methods Instrum Comput. (2004) 36:717–31. 10.3758/BF03206553 15641418

[24] Hanley JA McNeil BJ. The meaning and use of the area under a receiver operating characteristic (ROC) curve. Radiology. (1982) 143:29–36. 10.1148/radiology.143.1.7063747 7063747

[25] Boulouis G Morotti A Brouwers HB Charidimou A Jessel MJ Auriel E. Association between hypodensities detected by computed tomography and hematoma expansion in patients with intracerebral hemorrhage. JAMA Neurol. (2016) 73:961–8. 10.1001/jamaneurol.2016.1218 27323314 PMC5584601

[26] Thompson BB Bejot Y Caso V Castillo J Christensen H Flaherty ML. Prior antiplatelet therapy and outcome following intracerebral hemorrhage: a systematic review. Neurology. (2010) 75:1333–42. 10.1212/WNL.0b013e3181f735e5 20826714 PMC3013483

[27] Bochsen L Wiinberg B Kjelgaard-Hansen M Steinbruchel DA Johansson PI. Evaluation of the TEG platelet mapping assay in blood donors. Thromb J. (2007) 5:3. 10.1186/1477-9560-5-3 17311677 PMC1804261

[28] Schlunk F Greenberg SM. The pathophysiology of intracerebral hemorrhage formation and expansion. Transl Stroke Res. (2015) 6:257–63. 10.1007/s12975-015-0410-1 26073700

[29] Siddiqui TI Kumar KSA Dikshit DK. Platelets and atherothrombosis: causes, targets and treatments for thrombosis. Curr Med Chem. (2013) 20:2779–97. 10.2174/0929867311320220004 23590713

[30] Rincon F Mayer SA. Intracerebral hemorrhage: clinical overview and pathophysiologic concepts. Transl Stroke Res. (2012) 3:10–24. 10.1007/s12975-012-0175-8 24323860

[31] Al-Kawaz MN Hanley DF Ziai W. Advances in therapeutic approaches for spontaneous intracerebral hemorrhage. Neurotherapeutics. (2020) 17:1757–67. 10.1007/s13311-020-00902-w 32720246 PMC7851203

[32] Mayer SA Brun NC Begtrup K Broderick J Davis S Diringer MN. Efficacy and safety of recombinant activated factor VII for acute intracerebral hemorrhage. N Engl J Med. (2008) 358:2127–37. 10.1056/NEJMoa0707534 18480205

[33] Mrochen A Sprugel MI Gerner ST Sembill JA Lang S Lucking H. Thrombocytopenia and clinical outcomes in intracerebral hemorrhage: a retrospective multicenter cohort study. Stroke. (2021) 52:611–9. 10.1161/STROKEAHA.120.031478 33430632

[34] Magid-Bernstein J Beaman CB Carvalho-Poyraz F Boehme A Hod EA Francis RO. Impacts of ABO-incompatible platelet transfusions on platelet recovery and outcomes after intracerebral hemorrhage. Blood. (2021) 137:2699–703. 10.1182/blood.2020008381 33649761 PMC9635530

[35] Naidech AM Maas MB Levasseur-Franklin KE Liotta EM Guth JC Berman M. Desmopressin improves platelet activity in acute intracerebral hemorrhage. Stroke. (2014) 45:2451–3. 10.1161/STROKEAHA.114.006061 25005444 PMC6211162

[36] Kapapa T Rohrer S Struve S Petscher M Konig R Wirtz CR. Desmopressin acetate in intracranial haemorrhage. Neurol Res Int. (2014) 2014:298767. 10.1155/2014/298767 25610644 PMC4290038

[37] Gladstone DJ Aviv RI Demchuk AM Hill MD Thorpe KE Khoury JC. Effect of recombinant activated coagulation factor VII on hemorrhage expansion among patients with spot sign-positive acute intracerebral hemorrhage: the SPOTLIGHT and STOP-IT randomized clinical trials. JAMA Neurol. (2019) 76:1493–501. 10.1001/jamaneurol.2019.2636 31424491 PMC6704754

[38] Meretoja A Yassi N Wu TY Churilov L Sibolt G Jeng JS. Tranexamic acid in patients with intracerebral haemorrhage (STOP-AUST): a multicentre, randomised, placebo-controlled, phase 2 trial. The Lancet Neurology. (2020) 19:980–7. 10.1016/S1474-4422(20)30369-0 33128912

